# Genome-wide association analysis reveals genetic variations and candidate genes associated with salt tolerance related traits in *Gossypium hirsutum*

**DOI:** 10.1186/s12864-020-07321-3

**Published:** 2021-01-06

**Authors:** Peng Xu, Qi Guo, Shan Meng, Xianggui Zhang, Zhenzhen Xu, Wangzhen Guo, Xinlian Shen

**Affiliations:** 1 State Key Laboratory of Crop Genetics & Germplasm Enhancement, Hybrid Cotton R & D Engineering Research Center, Ministry of Education, Nanjing Agricultural University, Nanjing, 210095 China; 2 Provincial Key Laboratory of Agrobiology, Institute of Industrial Crops, Jiangsu Academy of Agricultural Sciences, Key Laboratory of Cotton and Rapeseed, Ministry of Agriculture and Rural Affairs, Nanjing, 210014 China

**Keywords:** *Gossypium hirsutum*, Genome-wide association study, Genotyping-by-sequencing, Salt tolerance, Virus-induced gene silencing assay

## Abstract

**Background:**

Cotton is more resistant to salt and drought stresses as compared to other field crops, which makes itself as a pioneer industrial crop in saline-alkali lands. However, abiotic stresses still negatively affect its growth and development significantly. It is therefore important to breed salt tolerance varieties which can help accelerate the improvement of cotton production. The development of molecular markers linked to causal genes has provided an effective and efficient approach for improving salt tolerance.

**Results:**

In this study, a genome-wide association study (GWAS) of salt tolerance related traits at seedling stage was performed based on 2 years of phenotype identification for 217 representative upland cotton cultivars by genotyping-by-sequencing (GBS) platform. A total of 51,060 single nucleotide polymorphisms (SNPs) unevenly distributed among 26 chromosomes were screened across the cotton cultivars, and 25 associations with 27 SNPs scattered over 12 chromosomes were detected significantly (−log_10_^p^ > 4) associated with three salt tolerance related traits in 2016 and 2017. Among these, the associations on chromosome A13 and D08 for relative plant height (RPH), A07 for relative shoot fresh matter weight (RSFW), A08 and A13 for relative shoot dry matter weight (RSDW) were expressed in both environments, indicating that they were likely to be stable quantitative trait loci (QTLs). A total of 12 salt-induced candidate genes were identified differentially expressed by the combination of GWAS and transcriptome analysis. Three promising genes were selected for preliminary function verification of salt tolerance. The increase of GH_A13G0171-silenced plants in salt related traits under salt stress indicated its negative function in regulating the salt stress response.

**Conclusions:**

These results provided important genetic variations and candidate genes for accelerating the improvement of salt tolerance in cotton.

## Background

The competition for arable land between food crops and cotton (*Gossypium* spp.) has existed for a long time in China. However, the emphasis on food security has inadvertently moved cotton production to more marginal soils in saline-alkali areas and coastal beaches due to the growing population. Cotton, the largest source of textiles fiber in the world is more resistant to abiotic stresses such as high salt and drought stresses than other crops. However, excessive salt in the soil can severely affect the growth and development of cotton plants [1], resulting in a reduction in fiber yield by as much as 60% [2]. Therefore, breeding cotton varieties with improved salt tolerance could alleviate the conflict between food crops and cotton by reclaiming and utilizing saline-alkali coastal lands for production. Similarly, in the northwestern inland cotton production region of China, the availability of salt tolerant varieties would expand the area of cotton production by promoting the synchronous growth of food crops and cotton.

The genetic architecture of salt tolerance is one of the most important subjects in plant science. Salt tolerance is a complex quantitative trait, which is controlled by multiple genes and involves a variety of physiological and biochemical metabolic pathways in cotton. In addition, the expression of each gene is sensitive to external environment. In the current study, the main conventional breeding approaches for development of salt tolerant varieties are screening and collecting salt tolerant germplasm resources, then transferring elite loci by hybridization, composite hybridization and backcrossing methods. The progress of conventional breeding of salt tolerance in upland cotton is impeded due to the lack of high salt tolerant genetic resources [3], low heritability [4], genetic complexity, and the difficulties in phenotyping [5].

The development of molecular markers linked to causal genes for a trait has provided an effective and efficient approach for improving quantitative traits. Once identified, markers linked to a quantitative trait locus (QTL) such as for salt tolerance can then serve as a selection tool for rapid and efficient marker-assisted selection (MAS). QTL mapping via bi-parental populations is an important method for quantitative trait research, and has been widely employed to map a number of traits including salt tolerance in various crops [6]. In addition to QTL mapping, association mapping based on linkage disequilibrium (LD) is another approach for detecting molecular markers tightly linked with quantitative traits in a natural germplasm population. Association analysis is time- and cost-effective, and can mine for genetic variations existed in the natural population, and more importantly, it takes advantage of the recombination information that has accumulated in the natural population over the long-term evolution process, thus achieving a higher mapping resolution, possibly identifying the causative genes [7, 8]. Because of its effectiveness in QTL identification, association mapping has been widely used in crop species and plays an important role in molecular breeding [9–14].

Genotyping-by-sequencing (GBS) is a reduced representation genotyping platform, and has a broad application in crop genetics and breeding [15–18]. In general, the GBS approach begins with an enzymatic digestion to reduce genome complexity using barcoding restriction enzymes, then performs multiple sequencing of barcoding DNA fragments on the high-throughput next-generation sequencing platform. A bioinformatics analysis of indexed sequence reads is followed to identify genetic variants. Finally, genetic diversity analysis is carried out based on a sample-by-variant matrix [19]. GBS is a powerful and cost-effective tool to assess variations across populations.

Since the first molecular marker-based genetic map of cotton published in 1994 [20], cotton scientists have identified a large number of QTLs regulating important agronomic traits, including fiber quality, yield, and disease resistance. However, only limited reports have been published on salt tolerance in cotton [21–25]. Herein, we reported the genome-wide association study (GWAS) of salt tolerant QTLs during the seedling stage performed over 2 years of phenotyping on 217 representative upland cotton cultivars. These results provided important genetic variations and candidate genes for accelerating the improvement of salt tolerance in cotton.

## Results

### Phenotypic diversity analysis

In order to evaluate the phenotypic variations of salt tolerance in the GWAS population with 217 upland cotton cultivars (Additional file 1: Table S1), three traits related to salt tolerance including relative plant height (RPH), relative shoot fresh matter weight (RSFW) and relative shoot dry matter weight (RSDW) were determined. The mean values, ranges, standard deviations (SD), coefficients of variation (CV) and broad-sense heritability (*H*^*2*^) for these salt tolerance related traits are shown in Table 1. Great differences of the CV in both years were found for the three traits. Overall, the upland cotton cultivars in this GWAS panel clearly exhibited considerable natural variations in the three traits to salt tolerance and displayed very high genetic diversity.

**Table 1:** Statistics of various traits related to salt tolerance

Trait	Range	Mean	SD	CV%	*H*^*2*^%
RPH16	0.189–0.760	0.440	0.145	0.330	92.802
RPH17	0.169–0.780	0.445	0.143	0.321	
RSFW16	0.136–0.762	0.411	0.150	0.365	91.873
RSFW17	0.129–0.794	0.420	0.167	0.398	
RSDW16	0.137–0.756	0.497	0.143	0.288	83.870
RSDW17	0.172–0.776	0.521	0.158	0.303	

*RPH16* Relative plant height in 2016, *RPH17* Relative plant height in 2017, *RSFW16* Relative shoot fresh matter weight in 2016, *RSFW17* Relative shoot fresh matter weight in 2017, *RSDW16* Relative shoot dry matter weight in 2016, *RSDW17* Relative shoot dry matter weight in 2017, *SD* Standard Deviation, *CV* Coefficient of Variation. H^2^: Broad-sense heritability

### Genetic diversity analysis

GBS produced 53,321 high-quality polymorphic single nucleotide polymorphisms (SNPs) among the 217 upland cotton cultivars, with 47,133 SNPs located in the intergenic intervals and 6188 SNPs in the coding regions. Of these, 95.8% of the loci (51,060 SNPs) were mapped onto 26 chromosomes of the cotton genome, and were selected for the GWAS analysis (Additional file 2: Table S2). These SNPs were not evenly distributed (Additional file 3: Fig.S1) with an average of 1964 SNPs per chromosome ranging from 863 to 5209. Chromosome A08 had the most SNPs (5209) with the highest SNP density (199 kb/SNP). D04 had the least SNPs (863), but A02 had the lowest SNP density with 680 kb/SNP. The genetic diversity of this population varied from 0.224 (A08) to 0.389 (A13). The polymorphism information content (PIC) varied from 0.192 (A08) to 0.305 (A13) (Table 2). The above results indicated a relatively large span in genetic diversity index and PIC in the cotton genome.

**Table 2:** Summary of polymorphic SNPs mapped in 26 chromosomes of *Gossypium hirsutum*

Chr.	Chr length (Mb)	No. of SNPs	SNP density (kb/SNP)	Gene diversity	PIC	LD Decay (kb) r^2^ = 0.1 r^2^ = 0.2	
A01	99.9	2111	473	0.294	0.235	1145	990
A02	83.5	1228	680	0.344	0.275	1215	903
A03	100.3	1490	673	0.321	0.26	1385	1035
A04	62.9	1138	553	0.314	0.255	1104	974
A05	92.1	2202	418	0.346	0.277	1354	1060
A06	103.2	3924	263	0.232	0.199	1377	1157
A07	78.3	1897	413	0.319	0.259	1258	959
A08	103.7	5209	199	0.224	0.192	1136	891
A09	75	1639	458	0.312	0.252	1299	1028
A10	100.9	2335	432	0.308	0.254	1191	957
A11	93.3	2027	460	0.257	0.215	1194	970
A12	87.5	1498	584	0.306	0.247	1183	915
A13	80	2996	267	0.389	0.305	1358	1058
D01	61.5	2385	258	0.318	0.261	880	435
D02	67.3	1914	352	0.321	0.26	712	599
D03	46.7	934	500	0.27	0.225	1046	803
D04	51.5	863	597	0.331	0.266	953	844
D05	61.9	1206	513	0.308	0.25	1204	816
D06	64.3	2454	262	0.281	0.235	1275	917
D07	55.3	2293	241	0.324	0.264	1124	817
D08	61.9	2463	251	0.306	0.248	909	523
D09	51	1643	310	0.286	0.239	1002	732
D10	63.4	1517	418	0.294	0.241	733	663
D11	66.1	1080	612	0.317	0.257	846	801
D12	59.1	1407	420	0.276	0.228	913	754
D13	60.5	1207	501	0.287	0.236	1325	988
Total	1931.1	51,060	427	0.303	0.248	1120	869

*Chr*. Chromosome, *PIC* Polymorphism information content

### Population structure and LD analysis

**Fig. 1:**
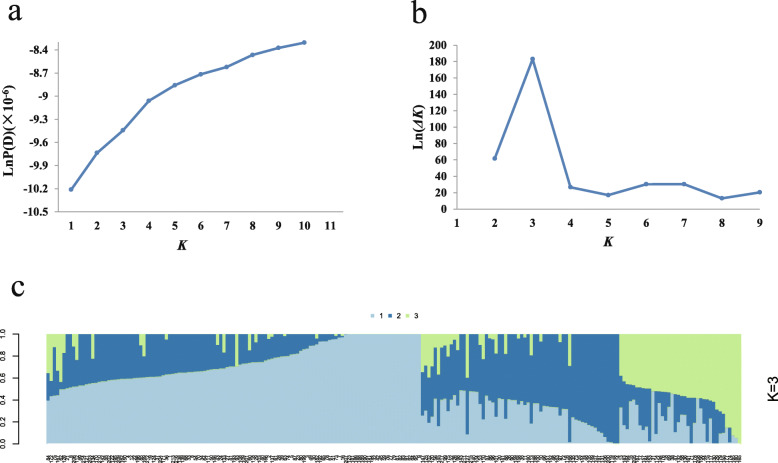
Population structure analysis of 217 cotton cultivars. **a** Estimated LnP(K) of possible clusters (K) from 1 to 10. **b** Delta K based on the rate of change of LnP(K) between successive K. **c** Population structure of 217 upland cotton accessions based on STRUCTURE when K = 3

It is important for GWAS analysis to control the effect of population structure, because population stratification could eliminate spurious associations between genotypes and phenotypes [26, 27]. STRUCTURE software was used to calculate the Bayesian clustering from K = 1 to 10 for five repetitions. LnP (D) value continued to increase from K = 1 to K = 10 without a significant inflection point (Fig. 1a). However, there was an obvious spike at the value of *Δ*K = 3 (Fig. 1b), suggesting that the population could be divided into 3 subgroups (Fig. 1c). Taking the corresponding Q matrix at k = 3 as the covariate could reasonably eliminate spurious association effects and improve the GWAS accuracy.

The LD distribution among chromosomes of the 217 upland cotton cultivars was shown in Table 2. The LD decay distance was 869 kb and 1120 kb when the r^2^ dropped to 0.2 and 0.1, respectively. The LD decay distance was not evenly distributed among chromosomes, ranged from 435 kb (D01) to 1157 kb (A06) when r^2^ was set at 0.2 and ranged from 712 kb (D02) to 1377 kb (A06) when r^2^ was set at 0.1, the overall LD decay in the At subgenome was significantly higher than that in the Dt subgenome.

### Association analysis

**Fig. 2:**
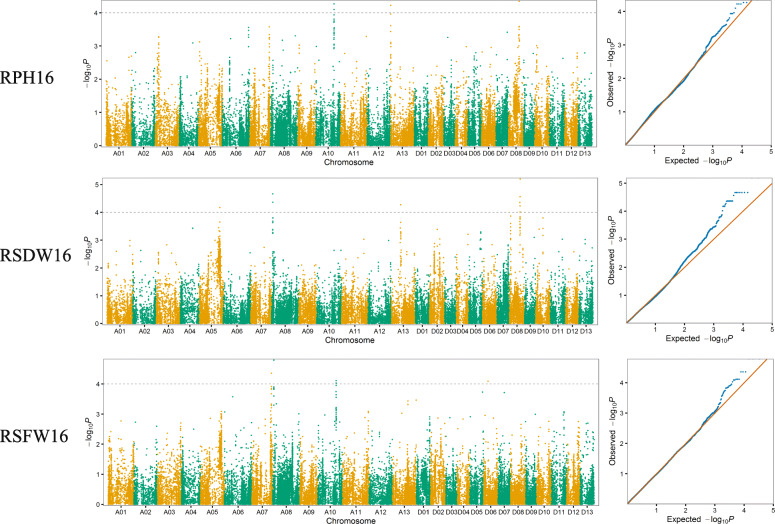
Manhattan and Q–Q plots for salt tolerance related traits in 2016. The horizontal dotted lines of the Manhattan plots with black color represent the genome-wide significance threshold of 0.0001

**Fig. 3:**
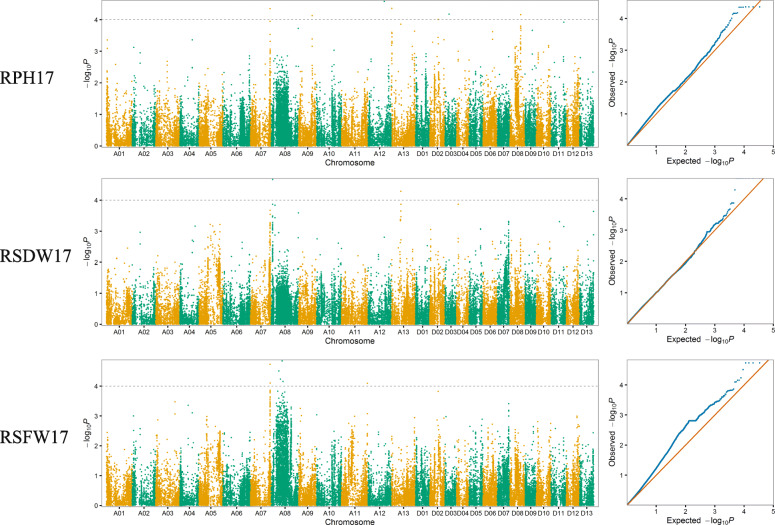
Manhattan and Q–Q plots for salt tolerance related traits in 2017. The horizontal dotted lines of the Manhattan plots with black color represent the genome-wide significance threshold of 0.0001

In order to explore the genetic factors underlying salt tolerance, the mixed linear models (MLMs) were performed by simultaneously accounting for population structure and relative kinship matrix to conduct a GWAS. A total of 25 significant associations (−log_10_^p^ > 4) with 27 significant SNPs located on chromosomes A05, A07, A08, A09, A10, A11, A12, A13, D02, D03, D06, and D09 were detected for the three salt tolerance related traits in the 2016 and 2017 dataset. Eleven associations with 12 SNPs were detected in 2016 (Fig. 2) and nine associations with 9 SNPs were detected in 2017 (Fig. 3). In addition, five associations with 6 SNPs were detected in both 2016 and 2017 dataset. The phenotypic variance explained (PVE) by individual QTL ranged from 1.29 to 7.00% (Table 3).

**Table 3:** Summary of SNPs significantly associated with salt tolerance related traits

Traits	Chr.	Site	Allele	MAF	-Log10(P)	PVE	Environment
RPH	A10	84,786,908	C/T	0.15(T)	4.27	5.67	2016
	A10	84,851,927	G/A	0.20(A)	4.10	4.31	2016
	A13	1,869,056	C/G	0.33(C)	4.22, 4.36	4.41,4.93	2016, 2017
	A07	90,682,411	C/T	0.12(T)	4.35	4.58	2017
	A09	62,356,818	A/G	0.19(A)	4.13	7.00	2017
	A12	74,098,493	G/A	0.49(A)	4.58	5.51	2017
	D02	38,449,756	T/A	0.47(T)	4.01	1.50	2017
	D03	18,961,034	T/G	0.11(G)	4.18	2.54	2017
	D08	49,014,753	C/T	0.13(T)	4.34, 4.16	4.35,3.83	2016, 2017
	D08	49,080,865	G/A	0.13(A)	4.34, 4.16	4.35,3.83	2016, 2017
RSFW	A08	5,515,194	C/T	0.10(T)	4.79	4.23	2016
	A10	84,786,908	C/T	0.15(T)	4.19	2.95	2016
	D06	19,966,743	C/G	0.5(G)	4.09	3.56	2016
	A07	90,532,061	C/G	0.13(G)	4.73	5.23	2017
	A07	90,682,411	C/T	0.12(T)	4.13, 4.73	2.40, 5.25	2016, 2017
	A08	41,804,418	T/A	0.09(T)	4.23	2.74	2017
	A08	53,916,411	G/A	0.088(G)	4.15	2.35	2017
	A11	117,938,337	G/A	0.32(A)	4.09	3.08	2017
RSDW	A05	94,406,377	C/T	0.33(T)	4.17	2.92	2016
	A08	5,589,045	G/A	0.19(A)	4.67	4.85	2016
	A08	6,254,890	T/G	0.22(G)	4.36,4.66	3.52,4.33	2016,2017
	A13	42,992,196	A/T	0.48(T)	4.27,4.28	2.79,3.03	2016,2017
	D08	47,703,450	C/T	0.096(T)	4.35	1.93	2016
	D08	48,527,413	C/A	0.12(A)	4.23	1.29	2016
	D08	48,911,757	C/G	0.13(G)	4.44	2.62	2016
	D08	49,014,753	C/T	0.13(T)	5.20	3.88	2016
	D08	49,080,865	G/A	0.13(A)	5.20	3.88	2016

*Chr.* Chromosome, *RPH* Relative plant height, *RSFW* Relative shoot fresh matter weight, *RSDW* Relative shoot dry matter weight, *MAF* Minor Allele Frequency, *PVE* Phenotypic variance explained. Rows with two values represent the two years data

For RPH, nine significant associations with 10 SNPs were identified on chromosomes A07, A09, A10, A12, A13, D02, D03, and D08. The PVE ranged from 1.50 to 7.00%. Moreover, the association with one SNP on chromosome A13 and the association with two SNPs on chromosome D08 were detected in both years.

For RSDW, eight significant associations with 9 SNPs on A05, A08, A13, and D08 were detected. The PVE ranged from 1.29 to 4.85%. The association with one SNP on chromosome A08 and the association with one SNP on chromosome A13 were detected in both years.

For RSFW, eight associations with 8 significant SNPs on A07, A08, A10, A11 and D06 were detected in 2016 and 2017. The PVE ranged from 2.35 to 5.25%. The association with three SNPs on chromosome A07 was detected in both years.

Pleiotropy was also found in our GWAS results. For example, the significant SNP A07_90,682,411 was simultaneously detected to be associated with RPH in 2017 and RSFW in both environments. The significant SNP A10_84,786,908 was simultaneously detected to be associated with RPH in 2016 and RSFW in 2016. The significant SNP D08_49,014,753 and D08_49,080,865 simultaneously detected to be associated with RPH in 2016, 2017, and RSDW in 2016. In addition, the associations on A08 for RSFW and RSDW in 2016 were only 70 kb apart. Their confidence interval overlapped with each other.

### Identification and preliminary function verification of candidate genes

**Fig. 4:**
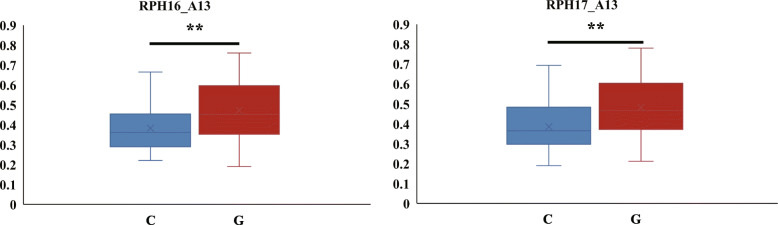
Box plots for the phenotypic values of the association for RPH on chromosome A13. ** indicate statistical significance at the 0.01 probability level. C: C haplotype. G: G haplotype

In general, the LD decay could be used as confidence interval to identify candidate genes. Because the cotton genome has a large LD decay [28, 29], we extracted potential candidate genes within 100 kb of flanking significant markers on the basis of the published upland cotton genome sequencing database [30]. A total of 156 genes were identified in these intervals (Additional file 4: Table S3). Gene ontology (GO) enrichment analysis indicated that “dioxygenase activity”, “oxidoreductase activity, acting on single donors with incorporation of molecular oxygen, incorporation of two atoms of oxygen” and “oxidoreductase activity, acting on single donors with incorporation of molecular oxygen” were significantly enriched using an false discovery rates (FDR) adjusted *P*-value of ≤0.05 as the cutoff. Of the 156 genes, 12 were differentially expressed between salt tolerant variety Miscott7913–83 and salt sensitive variety Su 12 according to the previous transcriptome sequencing results [31] (Additional file 4: Table S3). Some of these genes may be associated with salt stress, such as GH_A08G0488 and GH_A10G1620 encoding protein kinase. Protein kinase has been proved to play an important role in salt tolerance in cotton [32]. Another gene GH_A13G0171, which encodes aquaporins (AQPs), was also likely to regulate the salt stress response [33]. The confidence interval contains GH_A13G0171 was simultaneously detected in both years. We found that the salt tolerance of upland cotton cultivars with the G haplotype was significantly higher than that of cultivars with the C haplotype in both years upon a *t* test (Fig. 4).

**Fig. 5:**
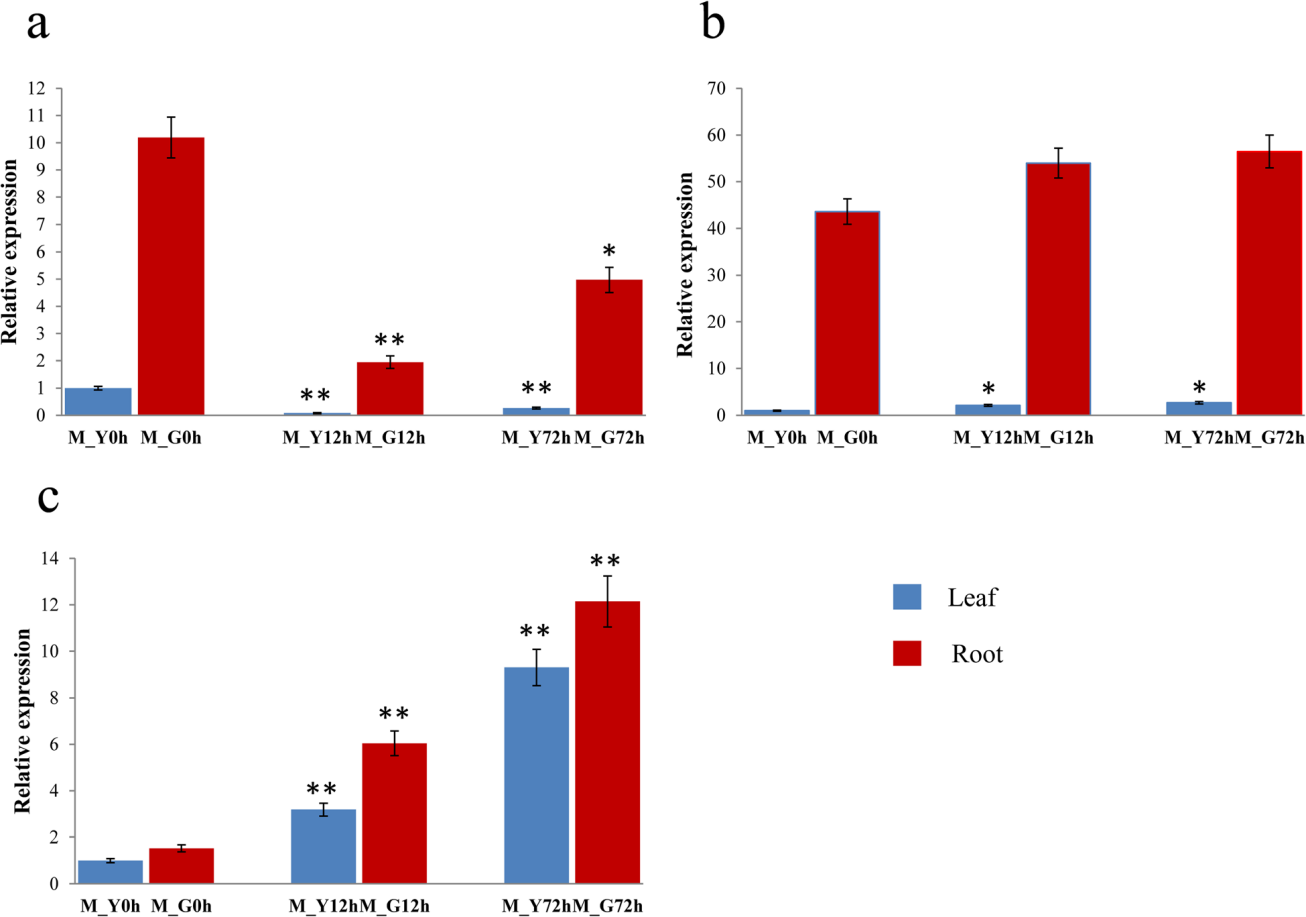
Expression profiles of three promising genes under NaCl stress at three time points in Miscott7913–83. **a** Expression profiles of GH_A13G0171. **b** Expression profiles of GH_A10G1620. **c** Expression profiles of GH_A08G0488. M: Miscott7913–83; Y: Leaf; G: Root. The samples were collected at 0 h, 12 h and 72 h under salt stress. The cotton *Actin* gene was used as an internal reference. The data are mean ± SE of three biological replicates. * and ** indicate statistical significance at the 0.05 and 0.01 probability level, respectively

**Fig. 6:**
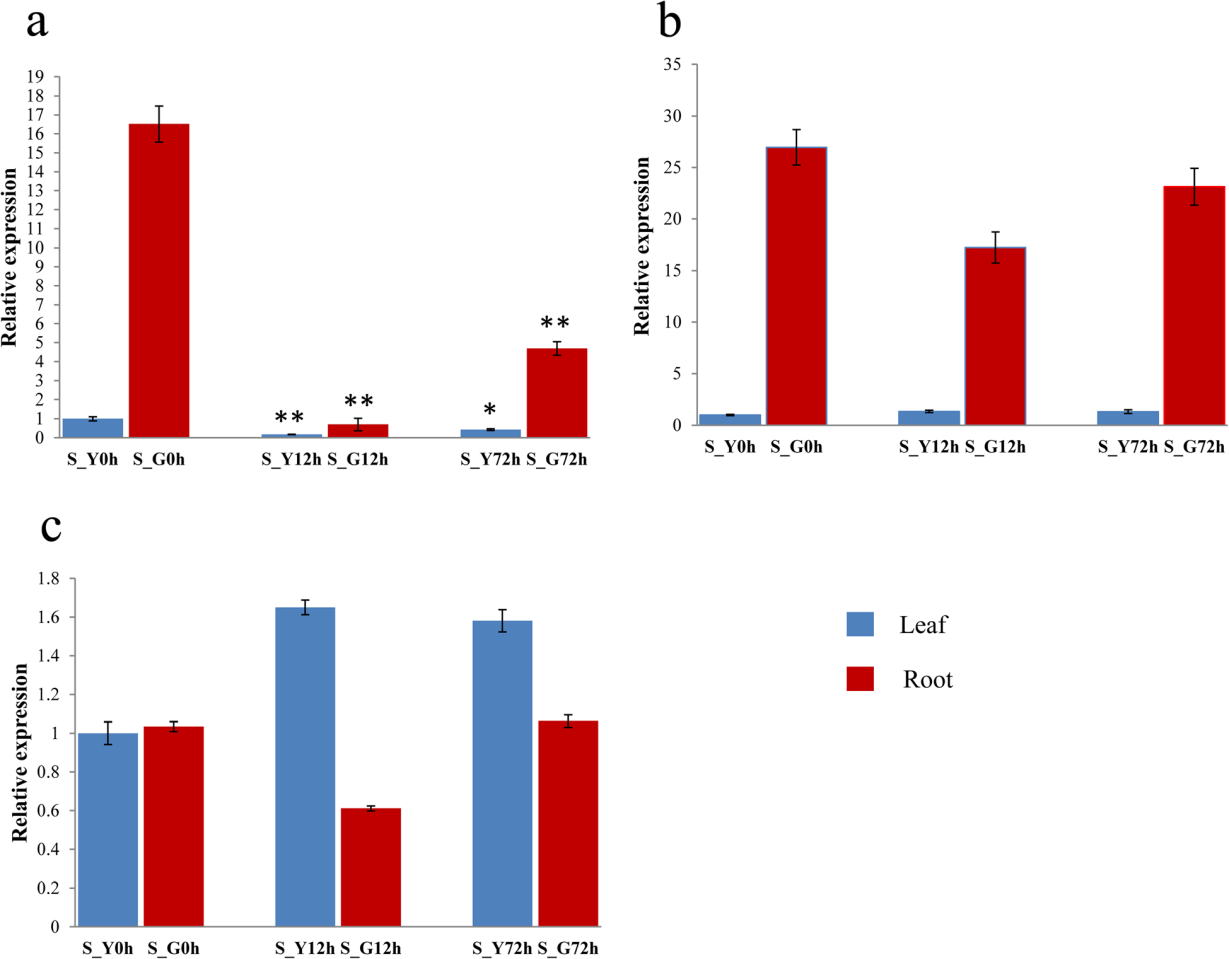
Expression profiles of three promising genes under NaCl stress at three time points in Su 12. **a** Expression profiles of GH_A13G0171. **b** Expression profiles of GH_A10G1620. **c** Expression profiles of GH_A08G0488. S: Su 12; Y: Leaf; G: Root. The samples were collected at 0 h, 12 h and 72 h under salt stress. The cotton *Actin* gene was used as an internal reference. The data are mean ± SE of three biological replicates. * and ** indicate statistical significance at the 0.05 and 0.01 probability level, respectively

The three promising genes (GH_A08G0488, GH_A10G1620 and GH_A13G0171) were selected for preliminary function verification of salt tolerance in cotton. Analysis of gene expression patterns could provide important clues for gene function determination. A quantitative real-time PCR (qRT-PCR) was performed to analyze the expression levels of GH_A08G0488, GH_A10G1620 and GH_A13G0171 in roots and leaves under salt stress treatment in salt tolerant variety Miscott7913–83 and salt sensitive variety Su 12. As shown in Fig. 5, the three genes were induced by salt stress and displayed distinct expression patterns in response to salt stress in salt tolerant variety Miscott7913–83. The three genes had a much higher expression level in roots than in leaves. The gene GH_A13G0171 exhibited a significantly down-regulated expression in both root and leaf tissues after salt stress. The gene GH_A08G0488 exhibited a significantly up-regulated expression in both root and leaf tissues. The expression level of GH_A10G1620 showed an increase in leaf and no significant changes in the root tissues. As shown in Fig. 6, the three genes were also displayed distinct expression patterns in response to salt stress in salt sensitive variety Su 12. The gene GH_A13G0171 exhibited an identical expression pattern in Miscott7913–83 and Su 12. The expression levels of GH_A10G1620 and GH_A08G0488 were not significant different.

**Fig. 7:**
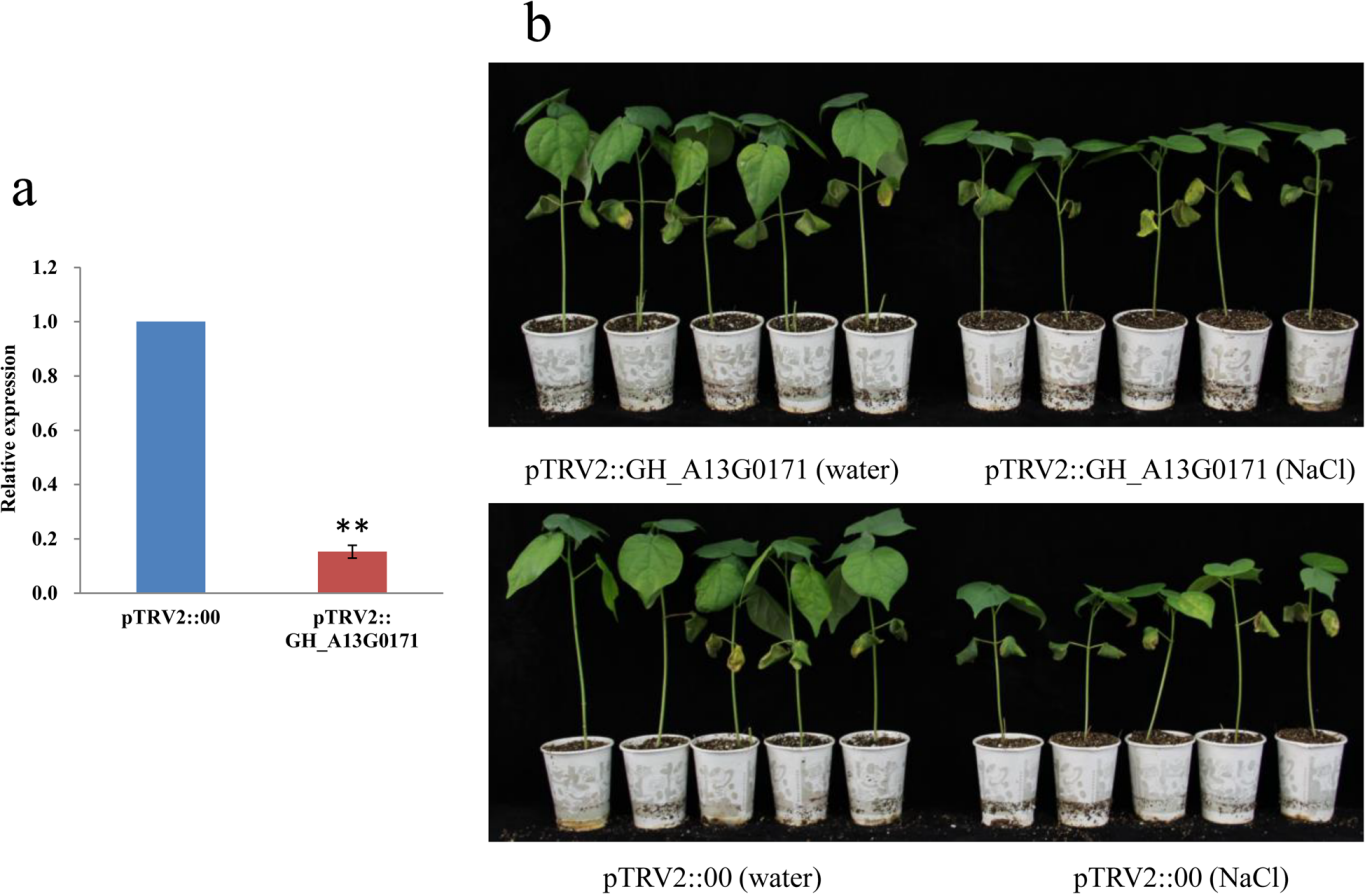
Functional characterization of GH_A13_G0171 VIGS in Miscott7913–83. **a** Transcript levels of GH_A13G0171 in pTRV2:: 00 and pTRV2:: GH_A13G0171 inoculated plants. The cotton *Actin* gene was used as an internal reference. The data are mean ± SE of three biological replicates. ** indicate statistical significance at the 0.01 probability level, respectively. **b** Phenotype identification of salt tolerance of the VIGS line and its control in seedling stage

To confirm the functional roles of GH_A08G0488, GH_A10G1620 and GH_A13G0171 genes under salt stress, virus-induced gene silencing (VIGS) assay was used to repress expression of these genes in salt tolerant variety Miscott7913–83 plants. The inoculated seedlings were grown in three light incubators at 23 °C under a 16-h light and 8-h dark cycle as three biological replicates. At the developmental period when two leaves had formed, the pTRV2:: GH_A08G0488, pTRV2:: GH_A10G1620, pTRV2:: GH_A13G0171 and pTRV2:: 00 inoculated plants were treated with 350 mM NaCl. After 15 days, the plant height, fresh and dry shoot matter weight were determined and the corresponding relative values were calculated. The transcripts of the three genes in the VIGS leaves were significantly reduced compared to the negative control pTRV2:: 00 inoculated plants, indicating that they were effectively silenced (Additional file 5: Fig.S2, Fig. 7a). Compared with the control pTRV2:: 00, no significance effect on their phenotypes was observed in pTRV2:: GH_A08G0488 and pTRV2:: GH_A10G1620 inoculated plants, and the plant height, fresh and dry shoot matter weigh of GH_A13G0171-silenced plants were significantly higher than that of pTRV2:: 00 inoculated plants (Table 4, Fig. 7b), indicating that GH_A13G0171 could reduce seedling tolerance to salt stress. The corresponding markers SNP_A13_1,869,056 could be applied for improvement of cotton salt tolerance.

**Table 4:** Statistics of traits related to salt tolerance for the VIGS lines and its control at the seedling stage

	RPH	RSFW	RSDW
pTRV2::00	0.512 ± 0.033	0.497 ± 0.027	0.534 ± 0.032
pTRV2::GH_A13G0171	0.591 ± 0.022^a^	0.614 ± 0.032^a^	0.657 ± 0.029^a^
pTRV2::GH_A08G0488	0.481 ± 0.029	0.474 ± 0.033	0.506 ± 0.036
pTRV2::GH_A10G1620	0.473 ± 0.026	0.494 ± 0.024	0.515 ± 0.030

The data are mean ± SE of three biological replicates. ^a^indicate statistical significance at the 0.05 probability level

pTRV2::00: Negative control; pTRV2::GH_A13G0171: GH_A13G0171 knockout seedlings; pTRV2::GH_A08G0488: GH_A08G0488 knockout seedlings; pTRV2::GH_A10G1620: GH_A10G1620 knockout seedlings

## Discussion

An ideal GWAS population should encompass sufficient phenotypic and genotypic diversity [34]. Thus, selection of a suitable panel with abundant genetic diversity is especially critical for GWAS analysis. In this study, the population panel consisted of 217 cultivars from different ecological regions (the Yellow River, the Yangtze River, the Northwestern inland region, the Northern China region, America and Australia) Phenotypic analysis showed that the salt tolerance related traits in two environments had wide variations and stable changing trends in different environments. Genotyping analysis revealed an average genetic diversity and PIC of 0.303 and 0.248, respectively. As a result, the 217 cotton accessions with broad geographic and genetic diversity coverage in our research provided sufficient detection power for GWAS analysis.

Recent years, association mapping as an important method for the identification of complex quantitative traits has been widely used in cotton for various traits including salt tolerance [35–40]. Jia et al. [21] identified 3 simple sequence repeats (SSR) markers significantly associated with the relative survival rate under salt stress through association mapping methods using 323 *G. hirsutum* germplasms. Du et al. [22] performed association analysis of 304 upland cotton cultivars and identified 95 significant associations for 10 salt tolerance related traits at the germination and seedling stages. These studies were conducted based on the SSR markers with lower mapping resolution and smaller genome coverage. Therefore, the genetic factors of salt tolerance related traits discovered with high-density SNP markers could provide more precise insight into salt tolerance in cotton than previous studies. Sun et al. [24] performed a GWAS analysis to identify marker-trait associations under salt stress using 713 upland cotton accessions through the Illumina Infinium CottonSNP63K array. A total of 23 SNPs were significantly associated with two salt-tolerance-related traits. All of the above reported associations were only detected in a single environment. QTL mapping of important agronomic traits of cotton has been carried out for nearly two decades, but few successful practicing of breeding new varieties by MAS was reported [41]. An important reason is the insufficient available information on multi-environmental detected QTLs. Salt tolerance as a complex quantitative trait controlled by multiple genes needs repeated phenotypic evaluations in multiple environments. In this study, a total of 25 significant associations with 27 SNPs scattered over 12 chromosomes were detected for salt tolerance related traits. Among these, the associations on chromosome A13 and D08 for RPH, A07 for RSFW, A08 and A13 for RSDW were expressed in both environments, indicating that they were likely to be stable QTLs. These results provided important genetic variations for accelerating the improvement of salt tolerance in cotton.

The comparison between our GWAS results and previous reports showed that most of the 25 associations were novel reported loci. The physical location of the SSR marker NAU2561 on A05 significantly associated with the relative malondialdehyde (MDA) content reported by Du et al. (2016) [22] was only 1.7 Mb from SNP_A05_ 94,406,377, which was significantly associated RSDW in 2016. The physical distance between BNL1231, which was reported by Du et al. (2016) [22] to be associated with relative superoxide dismutase (SOD) activity, and SNP_A11_117,938,337 which was associated with RSFW in 2017 was only 1 Mb. Considering larger LD decay distances in the cotton genome, it is reasonable to presume that these two loci could be the same loci.

Many studies have reported the positive effects of AQPs in improving plant tolerance to salt stresses. In this study, both gene expression and silencing analyses showed that GH_A13G0171, which encodes AQPs, was likely to regulate the salt stress response. AQPs play an important role in maintaining water homeostasis in the responses of plants to environmental stress by regulating water movement across vacuolar membranes [42]. Overexpression of *MaPIP2–7* in banana improved tolerance to drought, cold, and salt stresses [43]. Overexpression of *SpAQP1* promoted seed germination and root growth under salt stress in transgenic tobaccos [33]. The negative effect of AQPs also has been reported. Wang et al. [44] found that overexpression of *GsTIP2;1* in *Arabidopsis thaliana* depressed salt tolerance and dehydration stress. Plants with overexpressed *GsTIP2;1* exhibited higher dehydration rate, suggesting that this gene may mediate stress response by increasing water loss. GH_A13G0171 is homologous to *Arabidopsis* gene AT4G00430. The transgenic plants overexpressing *PIP1;4* (AT4G00430) displayed a rapid water loss under dehydration stress, which resulted in retarded germination and seedling growth under drought stress [45]. Our data showed that GH_A13G0171-silenced plants were significantly improved in salt tolerance related traits, indicating its negative effect in regulating the salt stress response. The exact role of this gene still needs further verification by RNA interference or clustered regularly interspaced short palindromic repeats (CRISPR) technology. Nonetheless, this study adds further credence to the relationship between AQPs and plant stress response.

Preliminary functional verification by VIGS assay suggested that GH_A08G0488 and GH_A10G1620 encoding protein kinase were irrelevant to salt tolerance. However, the real relationships between the two genes and salt tolerance still require verification of transgenic overexpression technology. Protein kinase which is widespread in plants plays an important role in signal transduction and is involved in response to a variety of plant hormones and environmental signals. In order to study its role in conferring abiotic stress tolerance, Zhao et al. [32] introduced cotton *GbRLK* gene into *Arabidopsis thaliana*. The results showed that *GbRLK* was involved in drought and high salinity stress by activating abscisic acid signaling pathway. The overexpression of *GbRLK* gene may reduce water loss by regulating stress response genes, thus improving stress resistance. He et al. [46] found that the salt-induced gene *TaGSK1* from wheat was a highly conserved serine/threonine protein kinase. Overexpression of *TaGSK1* in *Arabidopsis thaliana* decreased the osmotic pressure and Na^+^ content, and improved the salt tolerance. Overall, further molecular and functional analysis of GH_A08G0488 and GH_A10G1620 would advance our understanding of the molecular mechanisms underlying salt stress.

## Conclusions

It is of great significance to explore the genetic variants and the underlying candidate genes in upland cotton for salt tolerance improvement. In this study, we reported the GWAS analysis of salt tolerant QTLs during the seedling stage by GBS platform. A total of 25 associations with 27 SNPs scattered over 12 chromosomes were detected significantly associated with three salt tolerance related traits in two environments, in which five associations were simultaneously expressed in both environments. The VIGS assay revealed that GH_A13G0171, which encodes AQP PIP-type, negatively to regulates the salt stress response. All of our findings consistently suggest the identified candidate genes would be useful in salt tolerance breeding in cotton upon further validation with direct functional analyses which are underway.

## Methods

### Plant materials

A collection of 217 upland cotton cultivars (*Gossypium hirsutum* L.) were selected to assemble a GWAS panel in this study. These cultivars were collected from the Institute of Industrial Crops, Jiangsu Academy of Agricultural Sciences, kindly supplied by the Institute of Cotton Research, Chinese Academy of Agricultural Science (CRI-CAAS) and Cotton Research Center of Shandong Academy of Agricultural Sciences. Of these upland cotton cultivars, 162 were collected from China and 55 were introduced from other countries. Chinese cultivars were from four ecological areas, i.e. the Yellow River (73), the Yangtze River (62), the Northwestern inland region (23), and the early maturation area in Northern China (4).

In 2015, the 217 upland cotton cultivars were planted at the Lishui plant experiment station, Jiangsu Academy of Agricultural Sciences, China. Genomic DNA extraction referred to the method described by Paterson et al. (1993) [47]. The central natural open bolls of the 217 upland cotton cultivars were hand-harvested and ginned. The delinted seeds were then used for the subsequent determination of salt tolerance assay.

### Investigation of salt tolerance related traits

The cotton seeds of each cultivar were surface sterilized, transferred into 9 cm sterile Petri dishes on filter paper, and then wetted with 35 mL distilled water for germination. The Petri dishes were incubated in a growth chamber at 32 °C with no light. The germinated seeds were then grown in cups of dimension 220 ml with equal amounts of 1:1 mix of the matrix and vermiculite, and placed in 57 cm × 41 cm plastic pallets in a greenhouse. At the developmental period when two leaves had formed, the seedlings for each cultivar with the same growth vigor were divided into three groups with ten plants each. The first ten seedlings with two replications per cultivar were selected before treatment, and their plant height, fresh and dry shoot matter weight were determined as the 0-day control; the second ten seedlings with two replications were selected for 350 mM NaCl treatment, and the third ten seedlings with two replications were treated with clean water. After 7 days, the plant height, fresh and dry shoot matter weight of NaCl and clean water treated seedlings were determined respectively. The phenotype increments under 350 mM NaCl in 7 days was calculated using the following formula: (phenotypic effects value under salt stress after 7 days - phenotypic effects value at 0 day). The phenotype increments under clean water in 7 days was calculated using the following formula: (phenotypic effects value under well-watered control after 7 days - phenotypic effects value at 0 day). The ratio of the phenotype increments under 350 mM NaCl and clean water was considered as the relative values. The phenotype of three salt tolerance related traits were calculated using the following formula: (phenotype increments under 350 mM NaCl / (phenotype increments under clean water) × 100%.

We analyzed three traits related to salt tolerance in 2 years, including RPH, RSFW and RSDW. The average trait values in each year were used for phenotypic analysis and GWAS analysis in a single environment. Statistical analysis of the phenotype of salt tolerance related traits was performed by SPSS. *H*^*2*^ of each trait was calculated from variance components. The REML method of PROC VARCOMP in SAS/STAT software (SAS Institute Inc., Cary, NC, USA) was conducted to estimate the variance components.

### Library preparation and Illumina sequencing for GBS analysis

Firstly, a GBS pre-design experiment was performed. The type of enzymes and their fragments size were evaluated based on three criteria. (1) The number of tags must be appropriate. (2) The enzymatic tags must be evenly distributed. (3) Repeated tags must be avoided. Tags about 50 bp was selected to maintain the sequence depth uniformity of different fragments. The GBS library was constructed in accordance with the pre-designed scheme and referred to the method described by Fan et al. (2018) [48].

Then, pair-end sequencing was performed on the selected tags using an Illumina high-throughput sequencing platform Illumina NovaSeq. We generated a total of 5927 Gb high quality data with an average sequencing depth of approximately elevenfold. The sequences were sorted according to the barcodes. To make sure that reads were reliable and without artificial bias (low quality paired reads, which mainly resulted from base-calling duplicates and adapter contamination) in the following analyses. C scripts were conducted to processed raw fastq format reads through a series of quality control procedures as followed: (1) removing reads with unidentified nucleotides (N) ≥10%; (2) removing reads with > 50% bases having phred quality < 5; (3) removing reads aligned to the adapter more than 10 nucleotides.

### SNP detection and annotation

The clean reads were anchored to the cotton reference genome [30] using Burrows-wheeler aligner (BWA) [49]. The SAM tools software [50] was used to convert alignment files to BAM files. After alignment, the genomic variants (in GVCF format for each accession) were identified with the Haplotype caller module and the GVCF model by Genome analysis toolkit (GATK) software [51].. SNP was filtered by the perl script called ANNOVAR [52].

### Population structure and LD analysis

The population structure of our mapping panel was inferred from the GBS data with STRUCTURE software [53]. Five independent runs were performed; the number of populations (K) was set from 1 to 10; the burn-in time and Markov-chain Monte Carlo replication numbers was set to 10,000. The optimal K value was determined by comparing the LnP (D) and *Δ*k based on the rate of change in LnP (D) [54]. A Q-matrix produced by STRUCTURE listed the estimated membership coefficients in a cluster for the subsequent association analysis. The relative kinship matrix was calculated by SPAGeDi [55]. The software TASSEL 3.0 package was employed to estimate the LD parameters (r^2^) of adjacent loci within the same LD chromosome for each pair of SNP loci [56].

### Association mapping

The TASSEL 3.0 software package [56] was employed to construct association tests of salt tolerance related traits. The MLMs was performed by simultaneously accounting for multiple levels of Q-matrix and K-matrix according to the methods described by Yu et al. (2006) [57].

### Expression analysis of salt-induced genes

Total RNA was extracted using an improved CTAB method [58]. For qRT-PCR, PrimeScript RT Master Mix (Perfect Real Time) from TaKaRa (Dalian, China) was used to synthesize first-strand cDNA. The qRT-PCR program was performed on an ABI QuantStudio 5 RealTime PCR System. The qRT-PCR reaction system referred to the method described by Xu et al. (2017) [59]. qRT-PCR was carried out with three biological replicates. The cotton *Actin* gene was employed as a reference gene. The specific primers for qRT-PCR are listed in Additional file 6: Table S4.

### Virus-induced gene silencing assay

About 300–500 bp fragments overlapped the C-terminal and 3′ untranslated region (UTR) from 3 differentially expressed genes (GH_A08G0488, GH_A10G1620 and GH_A13G0171) were isolated in Miscott7913–83. The specific primers were listed in Additional file 6: Table S4. These fragments were sequenced and cloned into double enzyme (*Eco*RI and *Bam*HI) digested pTRV2, generating a new vector named pTRV2:: gene. The help plasmid pTRV1, pTRV2:: gene, VIGS positive control pTRV2:: CLA1 and negative control pTRV2:: 00 (empty vector) were introduced into *Agrobacterium tumefaciens* strain GV3101 by heat shock method. The VIGS assay was performed according to the protocol described by Gao et al. (2011) [60]. A ratio of 1:1 mixed pTRV1 and pTRV2:: gene were injected into the seedlings with mature cotyledons but without a visible true leaf via a syringe infiltration. The inoculated seedlings were grown in a light incubator at 23 °C under a 16-h light and 8-h dark cycle. Two weeks later, the inoculated plants RNA was isolated to detect whether the target gene was still expressed. For each gene, a total of 100 seedlings were knocked out for subsequent salt tolerance identification.

## Supplementary Information

**Additional file 1: Table S1.** Two hundred seventeen upland cotton cultivars in this study

**Additional file 2: Table S2.** Statistics of SNPs produced by GBS

**Additional file 3: Figure S1.** The distribution of SNPS on 26 chromosomes

**Additional file 4: Table S3.** One hundred fifty-six potential candidate genes within 100 kb of flanking significant markers

**Additional file 5: Figure S2.** Transcript levels of GH_A08G488 and GH_A10G1620 in pTRV2:: 00 and pTRV2:: gene inoculated plants.

**Additional file 6: Table S4.** The specific primers for qRT-PCR and Virus-induced gene silencing assay

## Data Availability

The datasets generated during the current study are available in the National Center of Biotechnology Information (NCBI, http://www.ncbi.nlm.nih.gov/) under the accession PRJNA681102.

## Abbreviations

GWAS: Genome-wide association study
GBS: Genotyping-by-sequencing
SNPs: Single nucleotide polymorphisms
RPH: Relative plant height
RSFW: Relative shoot fresh matter weight
RSDW: Relative shoot dry matter weight
QTLs: Quantitative trait loci
MAS: Marker-assisted selection
LD: Linkage disequilibrium
SD: Standard deviations
CV: Coefficients of variation
H^2^: Broad-sense heritability
PIC: Polymorphism information content
MLMs: Mixed linear models
PVE: Phenotypic variance explained
GO: Gene ontology
FDR: False discovery rates
AQPs: Aquaporins
qRT-PCR: Quantitative real-time PCR
VIGS: Virus-induced gene silencing
SSR: Simple sequence repeats
MDA: Malondialdehyde
SOD: Superoxide dismutase
CRISPR: Clustered regularly interspaced short palindromic repeats
BWA: Burrows-wheeler aligner
GATK: Genome analysis toolkit
UTR: Untranslated region

## References

[1] Qadir M, Shams M (1997). Some agronomic and physiological aspects of salt tolerance in cotton (*Gossypium hirsutum* L.). J Agron Crop Sci.

[2] Greenway H, Munns R (1980). Mechanisms of salt tolerance in nonhalophytes. Annu Rev Plant Physiol.

[3] Ye W, Liu J (1998). Identification technology on salt tolerance of cotton germplasm and its application. China Cotton.

[4] Tiwari RS, Picchioni GA, Steiner RL, Jones DC, Hughs SE, Zhang J (2013). Genetic variation in salt tolerance at the seedling stage in an interspecific backcross inbred line population of cultivated tetraploid cotton. Euphytica.

[5] Flowers TJ (2004). Improving crop salt tolerance. J Exp Bot.

[6] Ashraf M, Foolad MR (2013). Crop breeding for salt tolerance in the era of molecular markers and marker-assisted selection. Plant Breed.

[7] Zhang D, Song HN, Chen H, Hao DR, Wang H, Kan GZ (2014). The acid phosphatase-encoding gene *GmACP1* contributes to soybean tolerance to low-phosphorus stress. PLoS Genet.

[8] Chu S, Wang J, Zhu Y, Liu S, Zhou X, Zhang H (2017). An R2R3-type MYB transcription factor, *GmMYB29*, regulates isoflavone biosynthesis in soybean. PLoS Genet.

[9] Iquira E, Humira S, François B (2015). Association mapping of QTLs for sclerotinia stem rot resistance in a collection of soybean plant introductions using a genotyping by sequencing (GBS) approach. BMC Plant Biol.

[10] Nambeesan SU, Mandel JR, Bowers JE, Marek L, Ebert D, Corbi J (2015). Association mapping in sunflower (*Helianthus annuus* L.) reveals independent control of apical vs. basal branching. BMC Plant Biol.

[11] Zanke CD, Rodemann B, Ling J, Muqaddasi QH, Plieske J, Polley A (2017). Genome-wide association mapping of resistance to eyespot disease (*Pseudocercosporella herpotrichoides*) in European winter wheat (*Triticum aestivum* L.) and fine-mapping of *Pch1*. Theor Appl Genet.

[12] Zhao Y, Wang H, Chen W, Zhao P, Gong H, Sang X (2017). Regional association analysis-based fine mapping of three clustered QTL for *verticillium wilt* resistance in cotton (*G. hirsutum* L.). BMC Genomics.

[13] Rahimi Y, Bihamta MR, Taleei A, Alipour H, Ingvarsson PK (2019). Genome-wide association study of agronomic traits in bread wheat reveals novel putative alleles for future breeding programs. BMC Plant Biol.

[14] Thapa R, Tabien RE, Thomson MJ, Septiningsih EM (2020). Genome-wide association mapping to identify genetic loci for cold tolerance and cold recovery during germination in rice. Front Genet.

[15] Edae EA, Olivera PD, Jin Y, Poland JA, Rouse MN (2016). Genotype-by-sequencing facilitates genetic mapping of a stem rust resistance locus in *Aegilops umbellulata*, a wild relative of cultivated wheat. BMC Genomics.

[16] Navarro J, Wilcox M, Burgueño J, Romay C, Swarts K, Trachsel S (2017). A study of allelic diversity underlying flowering-time. Nat Genet.

[17] Wu X, Guo X, Wang A, Liu P, Wu W, Zhao Q (2019). Quantitative trait loci mapping of plant architecture-related traits using the high-throughput genotyping by sequencing method. Euphytica.

[18] Saxena RK, Kale S, Mir RR, Mallikarjuna N, Yadav P, Das RR (2020). Genotyping-by-sequencing and multilocation evaluation of two interspecifc backcross populations identify QTLs for yield-related traits in pigeonpea. Theor Appl Genet.

[19] Peterson GW, Dong YB, Horbach C, Fu YB (2014). Genotyping by-sequencing for plant genetic diversity analysis: a lab guide for SNP genotyping. Diversity.

[20] Reinisch AJ, Dong JM, Brubaker CL (1994). A detailed RFLP map of cotton, *Gossypium hirsutum* × *Gossypium barbadense*: chromosome organization and evolution in a disomic polyploid genome. Genetics.

[21] Jia Y, Sun J, Wang X, Zhou Z, Pan Z, He S (2014). Molecular diversity and association analysis of drought and salt tolerance in *Gossypium hirsutum* L. Germplasm. J Integr Agric.

[22] Du L, Cai C, Wu S, Zhang F, Hou S, Guo W (2016). Evaluation and exploration of favorable QTL alleles for salt stress related traits in cotton cultivars (*G. hirsutum* L.). PLoS One.

[23] Zhao YL, Wang HM, Shao BX, Chen W, Guo ZJ, Gong HY (2016). SSR-based association mapping of salt tolerance in cotton (*Gossypium hirsutum* L.). Genet Mol Res.

[24] Sun Z, Li H, Zhang Y, Li Z, Ke H, Wu L (2018). Identification of SNPs and candidate genes associated with salt tolerance at the seedling stage in cotton (*Gossypium hirsutum* L.). Front Plant Sci.

[25] Yasir M, He S, Sun G, Geng X, Pan Z, Gong W (2019). A genome-wide association study revealed key SNPs/genes associated with salinity stress tolerance in upland cotton. Genes.

[26] Stich B, Maurer HP, Melchinger AE, Frisch M, Heckenberger M, van der Voort JR (2006). Comparison of linkage disequilibrium in elite European maize inbred lines using AFLP and SSR markers. Mol Breed.

[27] Yang XH, Yan JB, Zheng YP, Yu JM, Li JS (2007). Reviews of association analysis for quantitative traits in plants. Acta Agron Sin.

[28] Sun Z, Wang X, Liu Z, Gu Q, Zhang Y, Li Z (2017). Genome-wide association study discovered genetic variation and candidate genes of fibre quality traits in *Gossypium hirsutum* L. Plant Biotechnol J.

[29] Huang C, Nie X, Shen C, You C, Li W, Zhao W (2017). Population structure and genetic basis of the agronomic traits of upland cotton in China revealed by a genome-wide association study using high-density SNPs. Plant Biotechnol J.

[30] Hu Y, Chen J, Fang L, Zhang Z, Ma W, Niu Y (2019). *Gossypium barbadense* and *Gossypium hirsutum* genomes provide insights into the origin and evolution of allotetraploid cotton. Nat Genet.

[31] Xu P, Yang Y, Guo Q, Zhang X, Xu Z, Shen X (2016). Development of EST-SSR and EST-InDel markers associated with salt tolerance in upland cotton. Cotton Sci.

[32] Zhao J, Gao Y, Zhang Z, Chen T, Guo W, Zhang T (2013). A receptor-like kinase gene (*GbRLK*) from *Gossypium barbadense* enhances salinity and drought-stress tolerance in *Arabidopsis*. BMC Plant Biol.

[33] Chang W, Liu X, Zhu J, Fan W, Zhang Z (2016). An aquaporin gene from halophyte *Sesuvium portulacastrum*, *SpAQP1*, increases salt tolerance in transgenic tobacco. Plant Cell Rep.

[34] Flint-Garcia SA, Thuillet AC, Yu JM, Pressoir G, Romero SM, Mitchell SE (2005). Maize association population: a high-resolution platform for quantitative trait locus dissection. Plant J.

[35] Nie X, Huang C, You C, Li W, Zhao W, Shen C (2016). Genome-wide SSR-based association mapping for fiber quality in nation-wide upland cotton inbreed cultivars in China. BMC Genomics.

[36] Fang L, Wang Q, Hu Y, Jia Y, Chen J, Liu B (2017). Genomic analyses in cotton identify signatures of selection and loci associated with fiber quality and yield traits. Nat Genet.

[37] Wang M, Tu L, Lin M, Lin Z, Wang P, Yang Q (2017). Asymmetric subgenome selection and cis-regulatory divergence during cotton domestication. Nat Genet.

[38] Dong C, Wang J, Chen Q, Yu Y, Li B (2018). Detection of favorable alleles for yield and yield components by association mapping in upland cotton. Genes Genomics.

[39] Ma J, Liu J, Pei W, Ma Q, Wang N, Zhang X (2019). Genome-wide association study of the oil content in upland cotton (*Gossypium hirsutum* L.) and identification of *GhPRXR1*, a candidate gene for a stable QTL *qOC-Dt5-1*. Plant Sci.

[40] Zhu G, Gao W, Song X, Sun F, Hou S, Liu N (2020). Genome-wide association reveals genetic variation of lint yield components under salty field conditions in cotton (*Gossypium hirsutum* L.). BMC Plant Biol.

[41] Guo WZ, Zhang TZ, Zhu XF, Pan JJ (2005). Modified backcross pyramiding breeding with molecular marker-assisted selection and its applications in cotton. Acta Agron Sin.

[42] Kapilan R, Vaziri M, Zwiazek JJ (2018). Regulation of aquaporins in plants under stress. Biol Res.

[43] Xu Y, Hu W, Liu J, Song S, Hou X, Jia C (2020). An aquaporin gene *MaPIP2-7* is involved in tolerance to drought, cold and salt stresses in transgenic banana (*Musa acuminate* L.). Plant Physiol Biochem.

[44] Wang X, Li Y, Ji W, Bai X, Cai H, Zhu D (2011). A novel *Glycine soja* tonoplast intrinsic protein gene responds to abiotic stress and depresses salt and dehydration tolerance in transgenic *Arabidopsis thaliana*. J Plant Physiol.

[45] Jiang JY, Lee SH, Rhee JY, Chung GC, Ahn SJ, Kang H (2007). Transgenic *Arabidopsis* and tobacco plants overexpressing an aquaporin respond differently to various abiotic stresses. Plant Mol Biol.

[46] He X, Tian J, Yang L, Huang Y, Zhao B, Zhou C (2012). Overexpressing a glycogen synthase kinase gene from wheat, *TaGSK1*, enhances salt tolerance in transgenic *Arabidopsis*. Plant Mol Biol Report.

[47] Paterson AH, Brubaker CL, Wendel JF (1993). A rapid method for extraction of cotton (*Gossypium spp*.) genomic DNA suitable for RFLP or PCR analysis. Plant Mol Biol Report.

[48] Fan L, Wang L, Wang X, Zhang H, Zhu Y, Guo J (2018). A high-density genetic map of extra-long staple cotton (*Gossypium barbadense*) constructed using genotyping-by sequencing based single nucleotide polymorphic markers and identification of fiber traits-related QTL in a recombinant inbred line population. BMC Genomics.

[49] Li H, Durbin R (2009). Fast and accurate short read alignment with burrows-wheeler transform. Bioinformatics.

[50] Li H, Handsaker B, Wysoker A (2009). The sequence alignment/map format and SAMtools. Bioinformatics.

[51] McKenna A, Hanna M, Banks E, Sivachenko A, Cibulskis K, Kernytsky A (2010). The genome analysis toolkit: a MapReduce framework for analyzing next-generation DNA sequencing data. Genome Res.

[52] Wang K, Li M, Hakonarson H (2010). ANNOVAR: functional annotation of genetic variants from high-throughput sequencing data. Nucleic Acids Res.

[53] Pritchard JK, Stephens M, Donnelly P (2000). Inference of population structure using multilocus genotype data. Genetics.

[54] Evanno G, Regaut S, Goudet J (2005). Detecting the number of clusters of individuals using the software STRUCTURE: a simulation study. Mol Ecol.

[55] Hardy OJ, Vekemans X (2002). SPAGEDi: a versatile computer program to analyse spatial genetic structure at the individual or population levels. Mol Ecol Notes.

[56] Bradbury PJ, Zhang Z, Kroon DE, Casstevens RM, Ramdoss Y, Buckler ES (2007). TASSEL: software for association mapping of complex traits in diverse samples. Bioinformatics.

[57] Yu J, Pressoir G, Briggs WH, Vroh Bi I, Yamasaki M, Doebley JF (2006). A unified mixed-model method for association mapping that accounts for multiple levels of relatedness. Nat Genet.

[58] Hu G, Yu S (2007). Extraction of high-quality total RNA in cotton leaf with improved CTAB method. Cotton Sci.

[59] Xu P, Gao J, Cao Z, Chee PW, Guo Q, Xu Z (2017). Fine mapping and candidate gene analysis of *qFL-chr1*, a fiber length QTL in cotton. Theor Appl Genet.

[60] Gao X, Britt RC, Shan L, He P (2011). Agrobacterium mediated virus-induced gene silencing assay in cotton. J Vis Exp.

